# Impaired activity of the fusogenic micropeptide Myomixer causes myopathy resembling Carey-Fineman-Ziter syndrome

**DOI:** 10.1172/JCI159002

**Published:** 2022-06-01

**Authors:** Andres Ramirez-Martinez, Yichi Zhang, Marie-Jose van den Boogaard, John R. McAnally, Cristina Rodriguez-Caycedo, Andreas C. Chai, Francesco Chemello, Maarten P.G. Massink, Inge Cuppen, Martin G. Elferink, Robert J.J. van Es, Nard G. Janssen, Linda P.A.M. Walraven-van Oijen, Ning Liu, Rhonda Bassel-Duby, Richard H. van Jaarsveld, Eric N. Olson

**Affiliations:** 1 Department of Molecular Biology and Hamon Center for Regenerative Science and Medicine, University of Texas Southwestern Medical Center, Dallas, Texas, USA.; 2 Department of Genetics,; 3 Department of Neurology, and; 4 Department of Oral and Maxillofacial Surgery, University Medical Center Utrecht, Utrecht, Netherlands.

**Keywords:** Muscle Biology, Monogenic diseases, Muscle, Neuromuscular disease

## Abstract

Skeletal muscle fibers contain hundreds of nuclei, which increase the overall transcriptional activity of the tissue and perform specialized functions. Multinucleation occurs through myoblast fusion, mediated by the muscle fusogens Myomaker (*MYMK*) and Myomixer (*MYMX*). We describe a human pedigree harboring a recessive truncating variant of the *MYMX* gene that eliminates an evolutionarily conserved extracellular hydrophobic domain of MYMX, thereby impairing fusogenic activity. Homozygosity of this human variant resulted in a spectrum of abnormalities that mimicked the clinical presentation of Carey-Fineman-Ziter syndrome (CFZS), caused by hypomorphic *MYMK* variants. Myoblasts generated from patient-derived induced pluripotent stem cells displayed defective fusion, and mice bearing the human *MYMX* variant died perinatally due to muscle abnormalities. In vitro assays showed that the human *MYMX* variant conferred minimal cell-cell fusogenicity, which could be restored with CRISPR/Cas9–mediated base editing, thus providing therapeutic potential for this disorder. Our findings identify *MYMX* as a recessive, monogenic human disease gene involved in CFZS, and provide new insights into the contribution of myoblast fusion to neuromuscular diseases.

## Graphical abstract

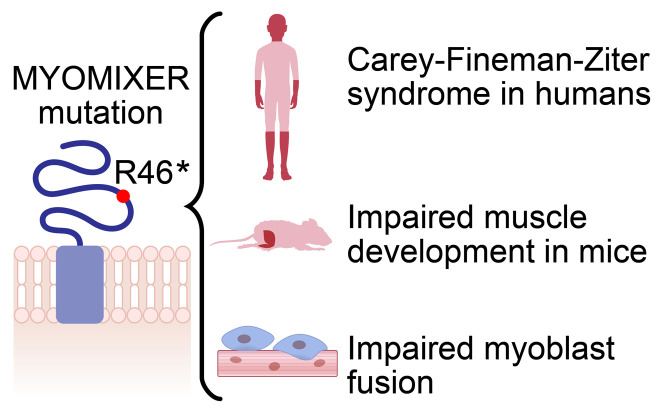

## Introduction

Cell-cell fusion is essential for numerous developmental events, including fertilization, placentation, and the formation of specialized cell types, such as osteoclasts and skeletal muscle fibers (reviewed in ref. 1). Intercellular fusion is a tightly regulated process, requiring recognition and merger of 2 or more cells to form a specialized multinucleated syncytium (2). As the largest tissue in the body, skeletal muscle undergoes extensive fusion during development as mononucleated myoblasts are recruited to nascent muscle fibers that ultimately expand to contain hundreds of nuclei (3). This process is essential for the establishment of the contractile apparatus and for motor innervation, allowing mobility.

Myoblast fusion involves a precisely coordinated series of events that include recognition between cells that are destined to fuse, followed by localization of fusogenic proteins to the sites of membrane mixing. We discovered 2 muscle-specific master regulators of myoblast fusion, Myomaker (4) (*Mymk*, also known as *Tmem8c*) and Myomixer (5–7) (*Mymx*, also known as Myomerger, Minion, or *Gm7325*), both of which are essential for muscle formation. MYMK is a 7-pass transmembrane protein that is required on both cells that are destined to fuse with each other (8), whereas MYMX is a single-pass transmembrane micropeptide that is required on only 1 of 2 fusing cells. When coexpressed in vitro, MYMX and MYMK are sufficient to promote fusion of cells that normally do not fuse, such as fibroblasts (5).

Fusogens juxtapose opposing membranes and expel the water between them, thus creating an energetically favorable environment for membrane merger, referred to as hemifusion, and the rapid opening of a fusion pore to mix cytosolic contents (9). Protein fusogens are typically characterized by long ectodomains capable of oligomerizing and reaching the opposing membrane upon conformational changes (10). Strikingly, MYMX is a small plasma membrane protein as short as 62 amino acids in some species (11). MYMX activity has been reported to be replaceable by osmotic shock in cell culture experiments (12), suggesting that MYMX may physically disrupt membrane integrity to drive fusion pore formation.

*Mymx* and *Mymk* are necessary for vertebrate skeletal muscle fusion in vivo, and mice lacking either of the 2 genes die at birth due to the lack of functional, multinucleated myofibers (4, 5). Similarly, deletion of *Mymx* or *Mymk* in adult satellite cells prevents skeletal muscle regeneration in response to injury (13, 14). These 2 fusogens are also conserved in zebrafish, where they are required for muscle formation (11, 15).

Whereas numerous muscle structural proteins have been associated with severe myopathies (16), relatively little is known about the potential contributions of the muscle fusion apparatus to human disease. Recently, hypomorphic variants in *MYMK* were shown to cause the congenital myopathy known as Carey-Fineman-Ziter syndrome (CFZS; OMIM #254940) (17–20). CFZS patients display an array of abnormalities, including hypotonia, myofiber size disproportion, Moebius sequence, Pierre Robin complex, and growth defects (21). Fibroblasts expressing *MYMK* CFZS variants fail to fuse in vitro, but these mutations do not fully prevent muscle formation in vivo (20).

Here, we report that a single-nucleotide variant (SNV) in *MYMX*, which results in the loss of the conserved extracellular hydrophobic ectodomain, is associated with a CFZS-like phenotype in humans. Loss of the MYMX ectodomain generates a stable protein truncation with impaired fusogenic activity. Disease modeling with skeletal muscle cells generated from patient-derived induced pluripotent stem cells (iPSCs) revealed impaired myoblast fusion in vitro, and mice bearing the human *MYMX* mutation died neonatally due to defects in muscle formation. Our findings identify *MYMX* as a human disease gene and reveal new insights into the molecular basis of muscle formation.

## Results

### Identification of a MYMX variant in myopathic patients.

**Figure 1:**
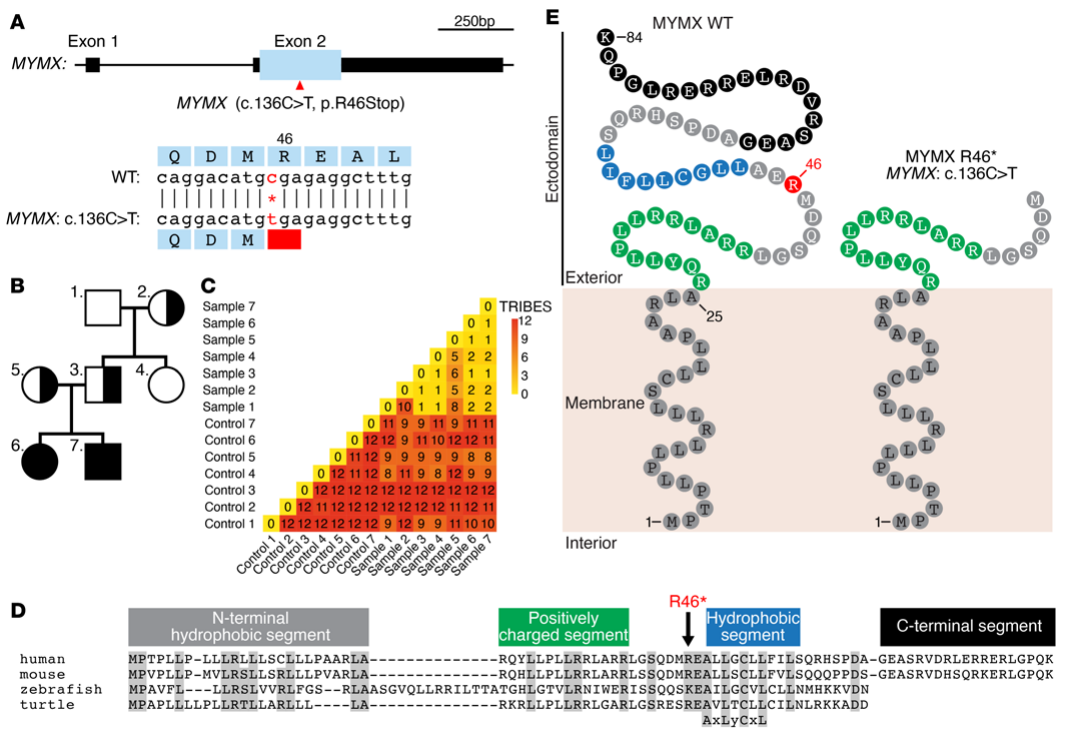
Identification of the *MYMX* R46* variant. (**A**) Top: Gene structure of human *MYMX*. Boxes represent exons. Black boxes denote untranslated regions, and blue box indicates open reading frame. Arrowhead indicates the location of the SNV (R46*) observed in myopathic patients. Scale of genomic length (bp) is shown at top right. Bottom: Genomic sequence surrounding *MYMX* R46*. (**B**) Pedigree of the family with the *MYMX* R46* variant. Squares indicate male; circles indicate female. Half filling indicates heterozygosity and full filling indicates homozygosity for *MYMX* R46*. Family members are assigned numbers used in **C**. (**C**) Identity-by-descent analysis to quantify relatedness. The TRIBES score indicates how genetically distant individuals are from each other. “Control” denotes unrelated individuals. “Sample” denotes family members from **B**. Sample 5 (mother) and sample 3 (father) showed a lower TRIBES score than unrelated controls, indicating distant relatedness. (**D**) Amino acid homologies among MYMX proteins from different species. (**E**) Predicted protein structure of MYMX WT and MYMX R46* variant.

Two individuals, a brother and a sister, presented in the clinic with a phenotype highly reminiscent of CFZS (22), a human disease caused by pathogenic variants in the *MYMK* gene (17–21). Diagnostic trio-exome sequencing of the affected patients did not identify any known pathogenic variants in myopathy-related genes, including *MYMK*. Open-exome analysis tailored toward the identification of recessive and/or de novo inherited variants revealed that both patients carried a homozygous C-to-T variant in codon 46 of *MYMX* on chromosome 6, resulting in conversion of Arg46 to a termination codon (NM_001315494.2 [*MYMX*]: c.136C>T [p.(Arg46*)]) (Figure 1A). Both parents were heterozygous carriers of this SNV (Figure 1B), which was considered a strong candidate to explain the phenotypes, as (a) the variant was absent from control genomes (gnomAD database; ref. 23), (b) loss-of-function mutations of *Mymx* in mice phenocopy loss-of-function mutations in *Mymk* (4, 5), and (c) the patients’ phenotype closely resembled CFZS, associated with recessive variants in *MYMK* (20). Identity-by-descent analysis (24) revealed that the parents were related multiple generations back, possibly indicating a founder effect for this specific variant (Figure 1C).

The human and mouse MYMX proteins contain 84 amino acids that include an N-terminal transmembrane hydrophobic segment followed by a positively charged extracellular segment and a shorter hydrophobic segment with a conserved AxLyCxL motif that is required for myoblast fusion (Figure 1D and ref. 5). Mammalian MYMX proteins also contain a hydrophilic C-terminal segment that is absent from fish and turtle proteins. The premature stop codon at position 46 (referred to hereafter as R46*) results in loss of most of the hydrophobic extracellular ectodomain of MYMX, including the essential AxLyCxL motif (Figure 1E). Therefore, we considered *MYMX* R46* a promising novel candidate genetic variant involved in CFZS.

**Figure 2:**
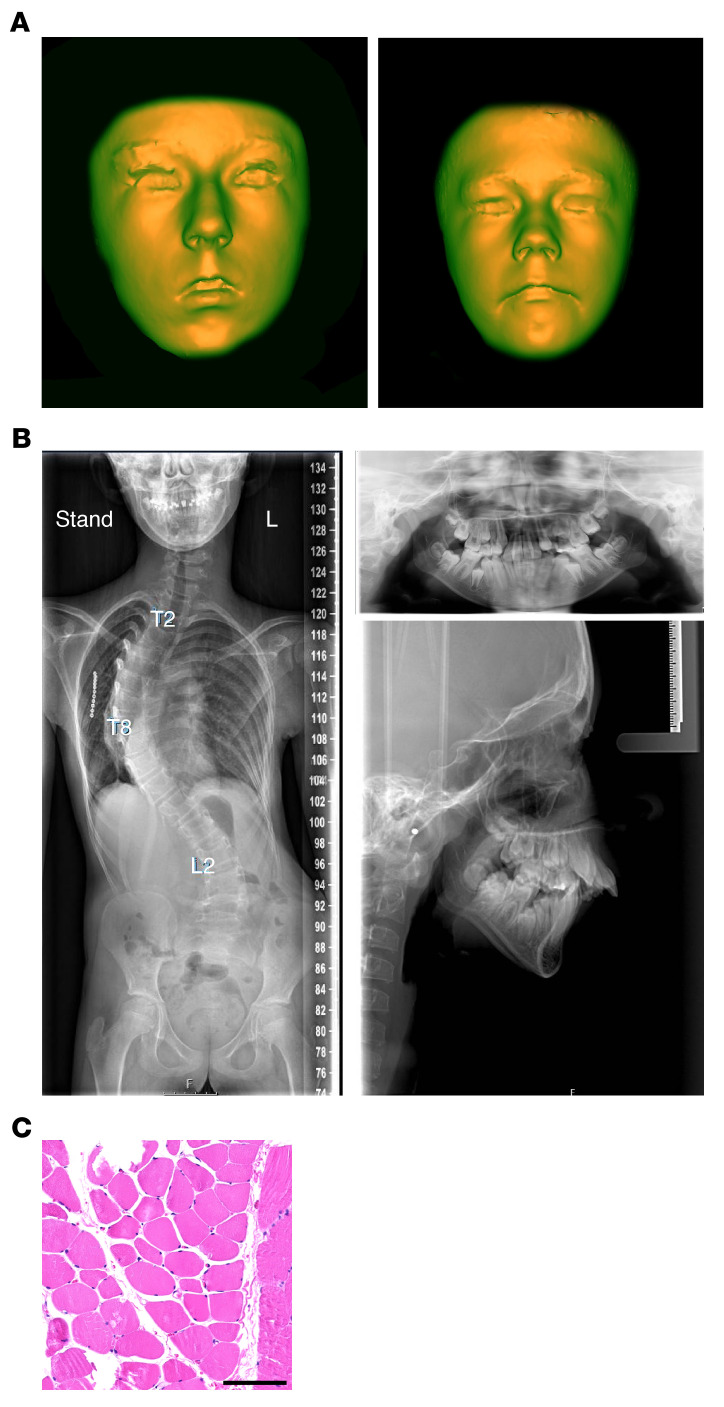
The *MYMX* R46* human phenotype resembles CFZS. (**A**) 3D reconstruction of facial dysmorphism in *MYMX* R46*/R46* female (left) and male (right) patients. Note that the hooded upper eyelid is more pronounced in reality than it appears on the reconstructions. (**B**) Radiography showing severe scoliosis (left) and mandibular abnormalities such as dental crowding and increased overbite (right) in a *MYMX* R46*/R46* patient. Thoracic (T) and lumbar (L) vertebrae are indicated. L, left. (**C**) H&E histology of musculus longissimus dorsi from a *MYMX* R46*/R46* patient. Scale bar: 100 μm.

Clinical presentations of both patients included weakness of the facial musculature, hypomimic face, increased overbite, micrognathia, and facial dysmorphism (Figure 2A), while their parents were unaffected (Table 1). The female patient appeared more severely affected, presenting with failure to thrive, axial hypotonia, and progressive scoliosis (Figure 2B and clinical report in the supplemental material; supplemental material available online with this article; https://doi.org/10.1172/JCI159002DS1). Prior studies showed fiber type disproportion and myopathy in quadriceps muscles from CFZS patients (20). However, a muscle biopsy taken from the trunk muscle (musculus longissimus dorsi) of the female patient showed minimal pathology (Figure 2C), probably reflecting differences across muscle groups. A muscle biopsy from the male patient was not available. Overall, these findings suggested that compromised MYMX activity could potentially cause muscle abnormalities resembling those of CFZS patients, who harbor hypomorphic *MYMK* variants.

### The MYMX R46* variant prevents fusion of myoblasts from patient-derived iPSCs.

**Figure 3:**
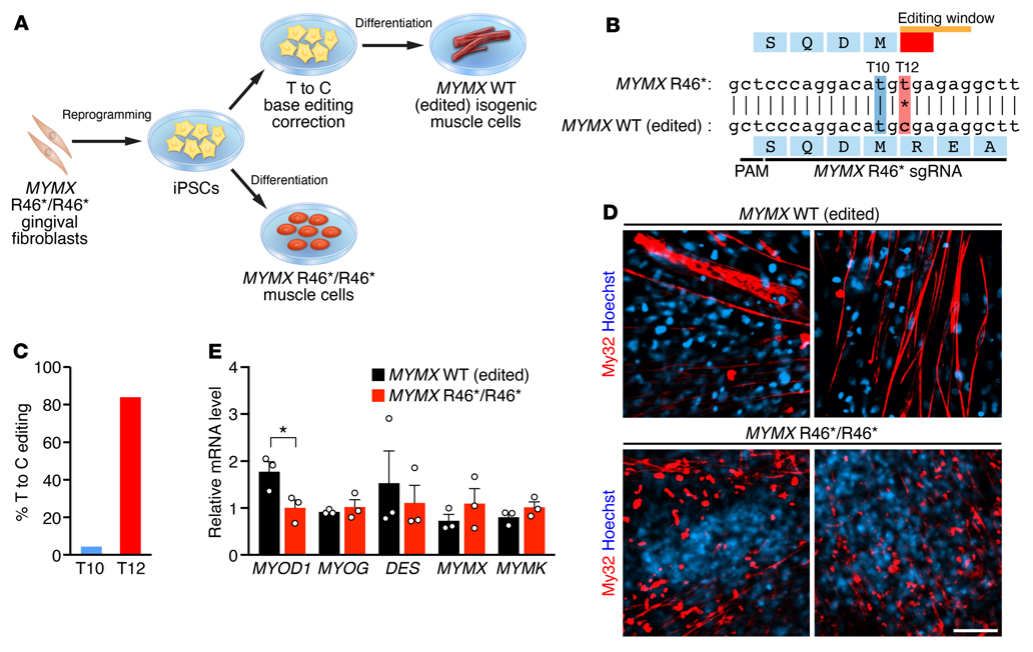
Modeling the *MYMX* R46* variant in skeletal muscle cells differentiated from patient-derived iPSCs. (**A**) Gingival fibroblasts from a male patient homozygous for the *MYMX* R46* variant were used to generate iPSCs, which were then induced to differentiate into skeletal muscle cells. To generate isogenic control cells, adenine base editing was used to edit T to C to obtain the wild-type genomic sequence in iPSCs. (**B**) Genomic sequence surrounding codon 46 of the human *MYMX* locus harboring the *MYMX* R46* variant (indicated by a black asterisk). The sequence of the sgRNA used for adenine base editing is shown, along with the PAM sequence. Adenine base editing results in conversion of the variant T to a C (T12, shown in red) and restoration of the open reading frame. The base editing window is shown in orange. A possible bystander nucleotide (T10) for adenine base editing is shown in blue. (**C**) Percentage of T-to-C base editing at T10 and T12 as determined by EditR analysis. (**D**) *MYMX* WT (edited) and *MYMX* R46*/R46* iPSC-derived skeletal muscle cells were stained for myosin heavy chain expression by My32 antibody and for nuclei with Hoechst 33342. Fusion was impaired in *MYMX* R46*/R46* muscle cells (bottom), whereas edited myoblasts formed multinucleated myotubes (top). Scale bar: 50 μm. (**E**) Expression of myogenic factors (*MYOD1*, *MYOG*), desmin (*DES*), and muscle fusogens (*MYMX*, *MYMK*) in iPSC-derived skeletal muscle cells as detected by qRT-PCR. *n =* 3 replicates per group. Statistical comparisons between groups were evaluated by unpaired and 2-sided Student’s *t* test. *P < 0.05. Error is expressed as SEM.

To assess muscle abnormalities in vitro, skeletal muscle cells were differentiated from iPSCs derived from gingival fibroblasts from the male patient (Figure 3A). To precisely determine the contribution of the *MYMX* R46* variant to the human phenotype and eliminate the influence of other genetic variants present in the patient, we generated an isogenic control cell line by genetically editing the *MYMX* R46* allele to *MYMX* WT sequence. To revert the c.136C>T SNV in *MYMX* R46*, we used CRISPR/Cas9–mediated adenine base editing, which converts A·T genomic base pairs into G·C base pairs (25). Specifically, to generate isogenic control cells, homozygous *MYMX* R46* iPSCs were nucleofected with plasmids encoding the adenine base editor NG-ABEmax (26) and an sgRNA targeting the R46* locus (Figure 3B). Sanger sequencing of nucleofected iPSCs showed an editing efficiency of 84% of the targeted nucleotide (T12) in sorted cells with minimal bystander editing of nucleotide T10 (Figure 3C). Patient-derived, isogenic, single iPSC clones homozygous for *MYMX* R46* or for *MYMX* WT (edited) were used for subsequent studies.

Cell lines were differentiated into skeletal muscle by defined chemical factors (27), and myoblast fusion was examined. Whereas the *MYMX* WT (edited) isogenic control muscle cells readily formed long multinucleated myotubes, fusion of homozygous *MYMX* R46* muscle cells was severely impaired (Figure 3D). Reduced multinucleation was not caused by a failure to differentiate, as cells were positive for the myosin heavy chain muscle marker My32, as detected by immunostaining (Figure 3D). Notably, quantitative reverse transcription PCR (qRT-PCR) analysis revealed that while *MYOD1* transcript levels were slightly downregulated in *MYMX* R46* myotubes, the myogenic regulator *MYOG*, the differentiation marker *DES*, and the muscle fusogens *MYMK* and *MYMX* were expressed at equivalent levels in *MYMX* R46* myotubes, despite their inability to fuse (Figure 3E).

### Mymx R46* mice recapitulate a lethal CFZS-like phenotype.

**Figure 4:**
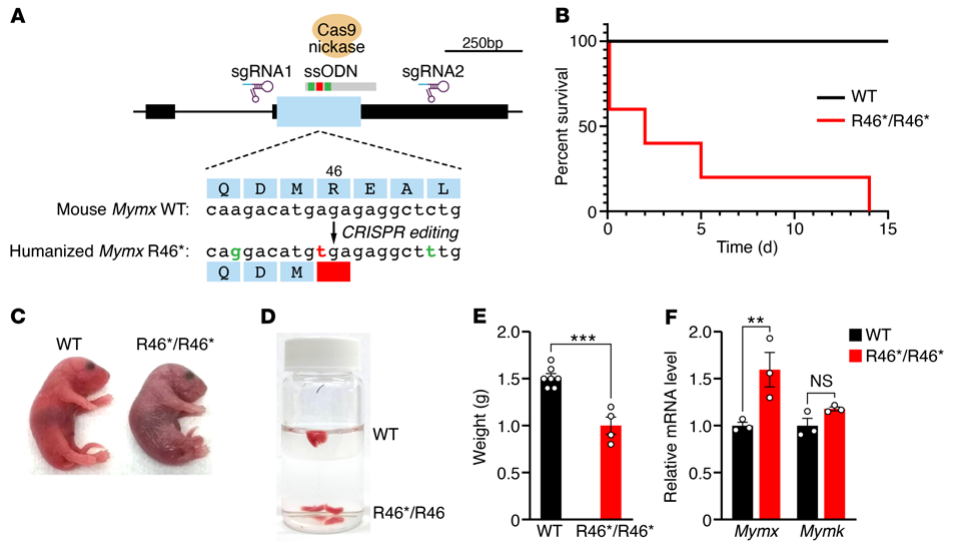
Modeling the *MYMX* R46* variant in mice. (**A**) The mouse *Mymx* gene showing positions of sgRNAs (sgRNA1 and sgRNA2) used for CRISPR-mediated knockin of *Mymx* R46* variant using Cas9 nickase. The nucleotide in red corresponds to the *Mymx* R46* variant, and the 2 nucleotides in green correspond to the mismatches between human and mouse sequences. The DNA template used was a single-stranded oligodeoxynucleotide donor (ssODN). Scale of genomic length (bp) is shown at top right. (**B**) Survival curve of WT and *Mymx* R46*/R46* mice. (**C**) Image of WT and *Mymx* R46*/R46* mice at birth. (**D**) Lung flotation assay. Lungs from *Mymx* R46*/R46* mice failed to inflate, whereas lungs from WT mice inflated and floated. (**E**) Body weight of WT and *Mymx* R46*/R46* mice at postnatal day 5. *n* = 7 (WT) and 4 (*Mymx* R46*/R46). ***P < 0.001. Error is expressed as SEM. (**F**) Gene expression of *Mymx* and *Mymk* in hind-limb muscles at birth. *n* = 3 animals per group. **P < 0.01. Statistical comparisons between groups were evaluated by unpaired and 2-sided Student’s *t* test. Error is expressed as SEM.

To model the muscle abnormalities associated with the *MYMX* R46* variant, we genetically engineered mice with this mutation by CRISPR/Cas9–mediated genome editing. The c.136C>T variant within codon 46 was introduced, together with 2 additional silent mutations, such that the surrounding genomic sequences were identical between human and mouse (Figure 4A). Heterozygous *Mymx* R46* mice showed no obvious abnormalities compared with WT mice. Homozygous *Mymx* R46*/R46* mice were born at the expected Mendelian ratios from heterozygous intercrosses. However, 40% of *Mymx* R46*/R46* mice died shortly after birth, and none survived beyond 14 days of age (Figure 4B). Mice that died at birth were clearly hypoxic (Figure 4C) and failed to inflate their lungs, as evidenced histologically (Supplemental Figure 1A) and by lung flotation assays (Figure 4D). The pups also lacked a milk spot in their stomach, indicative of a failure to feed. Surviving *Mymx* R46*/R46* mice showed postnatal growth defects, as seen by reduced body weight at postnatal day 5 (Figure 4E). The commercially available MYMX antibody cannot recognize MYMX R46*. However, gene expression analysis revealed increased levels of *Mymx* transcript in *Mymx* R46*/R46* hind-limb muscles, potentially as a compensatory mechanism, whereas *Mymk* expression remained unchanged between genotypes (Figure 4F).

**Figure 5:**
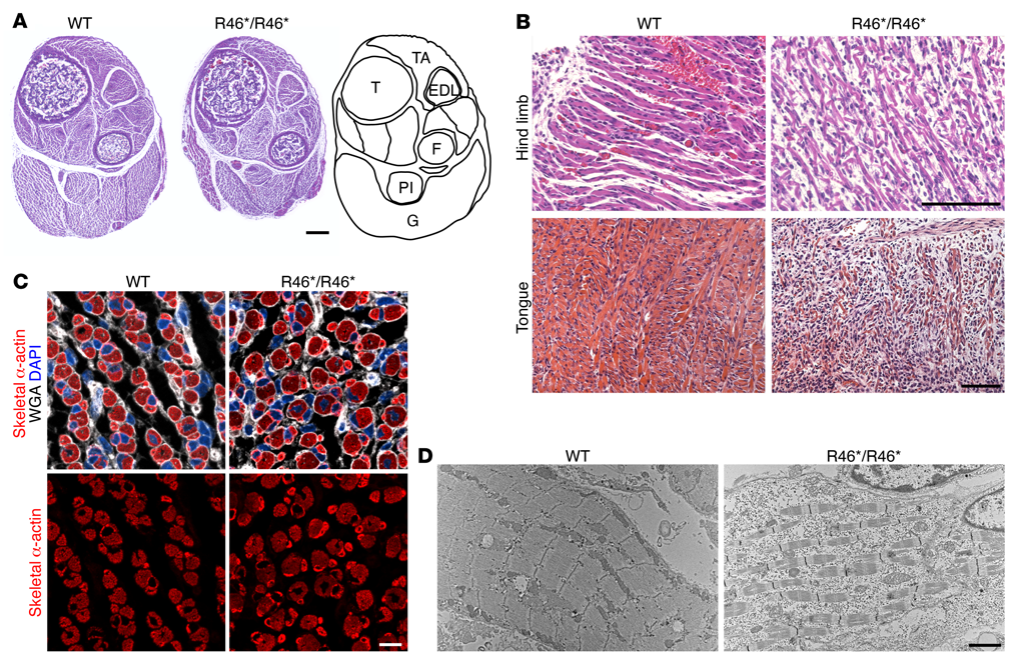
Defective myoblast fusion in *Mymx* R46*/R46* mice. (**A**) H&E staining of whole hind limbs from WT and *Mymx* R46*/R46* mice at birth. Muscle groups are present in *Mymx* R46*/R46* mice but are hypoplastic. An anatomic schematic is shown on the right. EDL, extensor digitorum longus; F, fibula; G, gastrocnemius; Pl, plantaris; T, tibia; TA, tibialis anterior. Scale bar: 100 μm. (**B**) H&E staining of transverse sections of hind-limb and tongue muscles of WT and *Mymx* R46*/R46* mice at birth. Scale bars: 100 μm. (**C**) Cross sections through gastrocnemius muscle of WT and *Mymx* R46*/R46* mice stained for skeletal α-actin (red), wheat germ agglutinin (WGA; white), and DAPI (blue). Muscle from *Mymx* R46*/R46* mice shows fiber size disproportion. Scale bar: 10 μm. (**D**) Hind-limb muscles from WT and *Mymx* R46*/R46* mice at birth were analyzed by electron microscopy. Whereas WT muscle showed highly organized sarcomeres, sarcomeres were sparse and highly fragmented in *Mymx* R46*/R46* muscle. Scale bar: 2 μm.

Histological analysis of hind limbs from WT and *Mymx* R46*/R46* mice revealed a reduction in muscle size across muscle groups (Figure 5A and Supplemental Figure 1B). Analysis of tongue and hind-limb muscles revealed severely impaired muscle fiber formation and fewer nuclei per myofiber in *Mymx* R46*/R46* mice that died at birth (Figure 5B), suggesting a hypomorphic fusion phenotype. Muscles from *Mymx* R46*/R46* mice also displayed fiber size disproportion (Figure 5C). Ultrastructural analysis of *Mymx* R46*/R46* hind-limb muscles by electron microscopy showed fragmented sarcomeres and evidence of myofiber degeneration (Figure 5D). Thus, the homozygous *Mymx* R46*/R46* mice recapitulated a myopathic phenotype reminiscent of that of CFZS patients.

### MYMX R46* is a stable hypomorphic protein with minimal fusogenicity.

**Figure 6:**
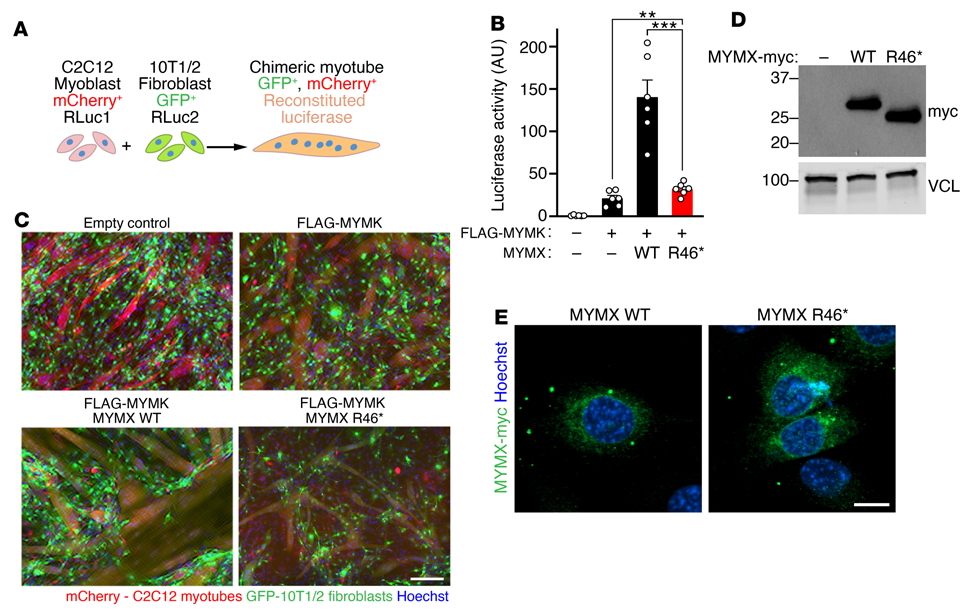
Functional analysis of the MYMX ectodomain in vitro. (**A**) Scheme for quantitative heterologous fusion assays. C2C12 myoblasts expressing mCherry and the first half of a split luciferase (RLuc1) were mixed with fibroblasts expressing GFP and the second half of split luciferase (RLuc2). Fusion can be observed by the presence of double-positive mCherry^+^GFP^+^ myotubes, and measured using cell-permeable luciferase substrates. (**B**) Luciferase measurements for heterologous fusion assays. Fibroblasts expressed the indicated constructs. *n =* 6 replicates per group. Statistical differences between groups were evaluated by 1-way ANOVA. **P < 0.01, ***P < 0.001. Error is expressed as SEM. (**C**) Representative images of heterologous fusion assays shown in **B**. Green corresponds to the GFP signal from fibroblasts, and red corresponds to the mCherry signal from myoblasts, so that chimeric myotubes are double positive (yellow). Nuclei were labeled with Hoechst 33342 (blue). Scale bar: 100 μm. (**D**) Western blot analysis of myc-tagged MYMX proteins, with MYMX WT and MYMX R46* showing similar protein levels. Vinculin (VCL) was used as a loading control. (**E**) Immunofluorescence of fibroblasts expressing the indicated myc-tagged MYMX constructs. All constructs were stable and show similar localization. Green indicates signal from myc staining. Nuclei were labeled with Hoechst 33342 (blue). Scale bar: 10 μm.

To understand the mechanistic basis of fusion abnormalities of the MYMX R46* variant, we generated retroviral constructs encoding full-length human MYMX WT or MYMX R46*. These constructs were functionally characterized in vitro with heterologous fusion assays (28), using a split luciferase system in which luciferase activity can be reconstituted by interaction of the N- and C-terminal domains of luciferase, referred to as RLuc1 and RLuc2, respectively (Figure 6A). For these assays, C2C12 myoblasts expressing mCherry-RLuc1 were mixed with 10T1/2 fibroblasts expressing GFP-RLuc2, and myoblast differentiation was induced. Myoblast-fibroblast fusion was then assessed by quantification of GFP^+^mCherry^+^ chimeric myofibers and measurement of reconstituted luciferase activity. In the presence of MYMK, MYMX WT strongly enhanced heterologous fusion, whereas the MYMX R46* variant had minimal fusogenicity (Figure 6, B and C). We then used C-terminally myc-tagged MYMX constructs to assess protein stability and localization. Western blot analysis (Figure 6D) and immunofluorescence of myc-tagged MYMX proteins (Figure 6E) revealed that MYMX WT and MYMX R46* constructs were expressed at equivalent levels and displayed comparable localization. These findings indicate that the *MYMX* R46* variant, observed in the CFZS-like patients, generates a stable truncated protein with similar localization to MYMX WT. However, truncation of the MYMX ectodomain diminishes its functional activity, impairing muscle fusion and causing a hypomorphic phenotype with developmental abnormalities associated with CFZS.

## Discussion

Myoblast fusion is essential for muscle development and postnatal muscle function (29). Here we show the involvement of *MYMX*, an essential muscle fusogen, in the etiology of CFZS, a progressive congenital myopathy with developmental defects (21). We identified 2 siblings presenting with CFZS clinical manifestations and revealed by trio-exome sequencing that they harbor a homozygous *MYMX* R46* variant. Prior studies on CFZS patients identified pathogenic gene variants in *MYMK*, another essential muscle fusogen, that caused protein instability and impaired muscle fusion (20). Further identification of other *MYMX* variants will help to decipher how muscle fusogens contribute to the pathology of CFZS and reconcile the diversity of histological phenotypes among patients. Overall, these studies indicate that defects in the master regulators of muscle fusion directly contribute to neuromuscular disorders, such as CFZS.

*MYMX* R46* is a truncated variant missing part of its extracellular region (ectodomain). Disease modeling of *MYMX* R46* using patient-derived iPSCs revealed defects in myoblast fusion, and in vitro fusion assays showed that the fusogenic activity of the MYMX R46* protein is severely compromised. The MYMX R46* protein is stable and shows no obvious abnormalities in localization. These results highlight the importance of the ectodomain of MYMX for myoblast fusion. It will be important to elucidate the precise molecular mechanism whereby this short domain drives cell fusion. Intriguingly, evolutionary analysis of MYMX orthologs revealed that the only region strictly conserved across species is the extracellular AxLyCxL motif (11), which is lost in the MYMX R46* truncation. In addition, the ectodomain of MYMX contains a stretch of basic residues that may interact with other charged molecules at the plasma membrane to promote fusion, and disruption of the MYMX ectodomain by combined point mutations is sufficient to abolish its fusogenic activity (5). Our data indicate that the MYMX R46* truncation is a pathogenic, hypomorphic variant, associated with impaired myoblast fusion that resembles the clinical manifestation of CFZS.

*Mymx* R46*/R46* mice represent a new mouse model for the human disorder, CFZS, caused directly by an impairment in myoblast fusion. *Mymx* R46*/R46* mice died shortly after birth, and histological analysis showed compromised myoblast fusion, whereas CFZS is not lethal in humans. The initial study that identified pathogenic *MYMK* variants associated with CFZS also included in vivo experiments in zebrafish (20) and reported that delivery of mRNAs encoding *MYMK* CFZS variants failed to rescue the *mymk*-null zebrafish model. Instead, those animals presented myopathic features. However, in contrast to *Mymk*-KO mice, *mymk*-null zebrafish are viable (30, 31). These phenotypic differences highlight the variability in myoblast fusion across species. For instance, in humans and mice, all muscle fibers undergo fusion, whereas in zebrafish, myoblast fusion only occurs in fast-twitch fibers (32). In vitro studies have shown that mouse *Mymx*-KO myoblasts cannot fuse, whereas in human *MYMX*-KO myoblasts, *MYMK* is sufficient to induce low levels of fusion (33). We hypothesize that additional genes may compensate for impaired *MYMX* function in humans, but not in mice. With the identification of *MYMK* and *MYMX* as muscle fusogens, evolutionary studies on myoblast fusion are necessary to further understand these differences.

The widespread role of cell-cell fusion in human disease has become increasingly appreciated. Defects in cell-cell fusion are involved in infertility (34), preeclampsia (35), osteoporosis (36), cancer (37), and infection (10, 38). In skeletal muscle, alterations in myoblast fusion have been reported to contribute to the pathology of human neuromuscular diseases (39, 40) such as Duchenne muscular dystrophy (41, 42), limb-girdle muscular dystrophy (43), and Emery-Dreifuss muscular dystrophy (44). The discovery of pathogenic variants in *MYMK* and *MYMX* highlights a new form of fusogenic myopathy. Myoblast fusion is required to increase the transcriptional diversity and capacity of skeletal muscle (45–48), and impairment of myoblast fusion below a certain threshold cannot be compensated by the remaining nuclei (42, 49, 50). It will be clinically important to determine whether genetic variants in other components of the fusion machinery cause similar human disorders.

Currently, there is no cure for CFZS. Gene therapy has been used successfully in clinical trials to treat genetic neuromuscular disorders such as spinal muscular atrophy (51, 52). However, *MYMX* expression is strictly restricted to myoblast fusion (5), and studies in mice suggest that overexpression of muscle fusogens could negatively impact muscle fiber integrity in dystrophic conditions (42). Recently, CRISPR/Cas9 base editing has emerged as an attractive therapeutic genome editing tool to directly correct disease-causing mutations and maintain endogenous expression levels of the corrected genes (53). Several studies have shown that in vivo delivery of base editors by adeno-associated viruses can target specific tissues, edit target genes, restore gene function, and ameliorate a broad spectrum of pathologies, including muscular dystrophies, metabolic disorders, and hereditary blindness (53). Using patient-derived iPSC myotubes, we show that the *MYMX* R46* variant is amenable to therapeutic correction by base editing. Further work with *Mymx* R46*/R46* mice and other humanized CFZS mouse models needs to be done to assess whether base editing can improve muscle pathology in vivo, and thus pave the way for a future treatment for this disorder.

## Methods

### Human genetic analyses.

The *MYMX* genetic variant was detected through clinical whole-exome sequencing (WES) performed at the University Medical Center Utrecht Genetics department according to local standardized diagnostic procedures. WES was performed using an Illumina NovaSeq 6000 platform on exome-enriched samples (Agilent Sureselect CREv2) from DNA isolated from peripheral blood. Diagnostic variant filtering based on the American College of Medical Genetics and Genomics/Association for Molecular Pathology guidelines (54) was performed using Agilent Alissa Interpret software. TRIBES software (v0.2.0) was used to estimate the degree of relatedness between samples (24). Analysis was performed on WES data from all family members and 7 randomly selected, unrelated in-house control samples. If no estimated degree of relatedness was detected, relatedness was manually set to 12.

### Generation of patient iPSC-derived skeletal muscle cells.

Gingival fibroblasts isolated from a *MYMX* R46*/R46* patient were reprogrammed into iPSCs with Sendai virus (Thermo Fisher Scientific, A16517) per the manufacturer’s instructions. Patient-derived iPSCs were maintained in mTeSR Plus medium (Stemcell Technologies, 100-0276) and plated on Matrigel-coated plates. iPSCs were dissociated with Accutase (Innovative Cell Technologies, AT104) and nucleofected with P3 Primary Cell 4D-Nucleofector X Kit (Lonza) per the manufacturer’s instructions. The plasmids used for nucleofection were NG-ABEmax (26) (Addgene, 124163) and pmCherry_gRNA (Addgene, 80457). The following sgRNA sequence was cloned into pmCherry_gRNA and used for base editing of the human *MYMX* locus: hs.MYMX.sgRNA: 5′-AGCCTCTCGCATGTCCTGGG-3′.

Forty-eight hours after nucleofection, mCherry-positive cells were isolated by FACS. The overall efficiency of base editing was assessed by isolation of genomic DNA from mCherry-positive cells, Sanger sequencing, and EditR analysis (55). Additionally, mCherry-positive single clones were isolated for genotyping and further studies. The following primers were used for PCR amplification with Taq polymerase (New England Biolabs, M0273): hs.MYMX.geno-F: 5′-GTGAGGCAGAACCAGGACAT-3′; hs.MYMX.geno-R: 5′-AACCTCTCCCTCCTCTCCAG-3′.

iPSCs were differentiated into skeletal muscle cells by defined factors as previously described (27). Briefly, the method recapitulates muscle development by the stepwise differentiation of iPSCs into paraxial mesoderm, myogenic progenitors, and, finally, muscle fibers.

### Immunofluorescence.

Cells were fixed for 10 minutes in 4% paraformaldehyde, permeabilized for 15 minutes in 0.3% Triton X-100, and blocked for 30 minutes in 5% BSA in PBS. The following antibodies were diluted in blocking solution at 1:200 dilution and added to cells for 1 hour: My32 (MilliporeSigma, M4276) and myc (Thermo Fisher Scientific, R950-25). Hoechst 33342 (Thermo Fisher Scientific, H3570) was used to label nuclei.

### Generation of Mymx R46* mice.

To generate humanized *Mymx* R46* mice, Cas9 nickase D10A was used (56). The sgRNAs used for injection were: mm.Mymx.sgRNA-5′: 5′-CAGAGCCTCTCTCATGTCTT-3′; mm.Mymx.sgRNA-3′: 5′-CCTCAGCCAGCAACAGCCAC-3′.

The single-stranded oligodeoxynucleotide donor (ssODN) used for injection is indicated below. Boldface denotes the SNV (R46*), and underline denotes additional silent mutations that humanize the locus and prevent recutting by Cas9: mm.Mymx.R46*-ssODN: 5′-CCACTCTGGAGGCCTCTCCAGAATCCGGTGGCTGTTGCTGGCTGAGGACAAAGAGCAGACAGCTCAGCAAAGCCTCTC**A**CATGTCCTGGGAGCTCAGTCGGCGGGCCAGCCGGC-3′.

Cas9 nickase D10A mRNA, sgRNAs, and ssODN were injected into the pronucleus of zygotes and transferred into the oviducts of pseudopregnant ICR female mice. F_0_ mice were genotyped by Sanger sequencing after genomic DNA isolation by phenol-chloroform purification. Taq polymerase (New England Biolabs, M0273) and the following primers were used for PCR amplification: mm.Mymx.geno-F: 5′-GCGTGCCTGAGGTACAGTCT-3′; mm.Mymx.geno-R: 5′-GTCAGAGCCCTCTTGCACTC-3′.

F_0_ mosaic mice were mated to C57BL/6N mice, and the progeny were genotyped to establish a mouse colony.

### Histological analysis.

Skeletal muscles and lungs were fixed for 16 hours at 4°C in 4% paraformaldehyde, and routine paraffin embedding and H&E staining were performed. For immunofluorescence, antigen retrieval was performed with SignalStain Citrate Unmasking Solution (Cell Signaling Technology, 14746) per the provider’s instructions. Sections were then permeabilized in 0.3% Triton X-100 for 15 minutes and blocked with mouse-on-mouse blocking solution (Vector Laboratories, BMK-2202) and 5% goat serum. The following antibodies were used at 1:200 dilution: ACTA1 (Proteintech, 17521-1-AP), wheat germ agglutinin and Alexa Fluor 647 conjugate (Thermo Fisher Scientific, W32466), and anti-rabbit Alexa Fluor 555 secondary antibody (Thermo Fisher Scientific, A32732). A Zeiss LSM 800 was used for image acquisition.

### Electron microscopy.

Skeletal muscles from neonatal mice were isolated and quickly fixed in 1% glutaraldehyde in 0.1 M sodium cacodylate (pH 7.4) and cut into small pieces. Staining was performed with 1% osmium tetroxide. Images were acquired on a JEOL 1400 Plus microscope. Samples were processed by the University of Texas Southwestern Medical Center Electron Microscopy Core facility.

### Gene expression analysis.

Hind-limb muscles from neonatal mice were flash-frozen and homogenized in 1 mL of TRIzol (Thermo Fisher Scientific, 15596026). Three mice per genotype were used. RNA isolation and RNA sequencing library preparation were performed with Quick-RNA Miniprep Plus Kit (Zymo Research, R1057) and KAPA mRNA HyperPrep kit (Kapa Biosystems, KK8580) per the providers’ instructions. RNA sequencing was performed by the CRI Sequencing Facility at the University of Texas Southwestern Medical Center. Bioinformatics analysis was performed as previously described (57). Low-quality reads were excluded (with fewer than 30% nucleotides with Phred quality scores below 20), and the remaining reads were aligned to the mouse genome (GRCm38.mm10) with HISAT2 aligner (v2.1.0) and counted with featureCounts (v1.6.2). DESeq2 R Bioconductor was used to obtain the differentially expressed genes between groups.

RNA from iPSC-derived skeletal muscle cells was solubilized in 1 mL of TRIzol (Thermo Fisher Scientific, 15596026), and cDNA was generated with iScript Reverse Transcriptase (Bio-Rad, 1725035). The following primers were used for qRT-PCR (33): hs.qPCR.MYOD1-F: 5′-CGACGGCATGATGGACTACA-3′; hs.qPCR.MYOD1-R: 5′-TATATCGGGTTGGGGTTCGC-3′; hs.qPCR.MYOG-F: 5′-GGGGAAAACTACCTGCCTGTC-3′; hs.qPCR.MYOG-R: 5′-AGGCGCTCGATGTACTGGAT-3′; hs.qPCR.MYMX-F: 5′-CTGATTCTGAGCAGCAGTTCT-3′; hs.qPCR.MYMX-R: 5′-AATGAACAGCAGACAGCCCA-3′; hs.qPCR.MYMK-F: 5′-TGTGCGGATCTACCATGACC-3′; hs.qPCR.MYMK-R: 5′-GACGCTCTTGTCTGGGTACAG-3′.

### Western blot analysis.

Protein was isolated from cell pellets by solubilization in RIPA buffer (MilliporeSigma, R0278). Bicinchoninic acid assay (Thermo Fisher Scientific, 23225) was used to determine protein concentration. Blocking and antibody incubation was performed in 5% milk, 0.1% Tween in TBS. The following antibodies were used at 1:200 dilution: myc (Thermo Fisher Scientific, R950-25) and vinculin (MilliporeSigma, V9131).

### In vitro fusion assays.

Quantitative heterologous fusion assays were described in prior studies (28). All cells were grown in DMEM supplemented with 10% FBS (Thermo Fisher Scientific, 26140087) and 1% antibiotic-antimycotic (Thermo Fisher Scientific, 26050088). Stable cell lines expressing the components for heterologous assays were generated by retroviral infection. Viral particles were generated in Platinum-E ecotropic cells (Cell Biolabs, RV-101) by transfection of retroviral plasmids with FuGENE6 (Promega, E2692) per the provider’s instructions. Viruses were filtered through a 0.45 μm membrane and concentrated with Retro-X concentrator (Takara, 631456). Polybrene (MilliporeSigma, TR-1003-G) was used for infection at a concentration of 8 μg/mL.

Retroviral infection was used to generate a stable C2C12 myoblast cell line (ATCC, CRL-1772) expressing mCherry and the first half of a split luciferase protein (RLuc1) and well as a stable 10T1/2 fibroblast cell line (ATCC, CCL-226) expressing GFP and the second half of the split luciferase protein (RLuc2). 10T1/2-GFP-RLuc2 fibroblasts were infected again with MYMK and MYMX to promote heterologous fusion. C2C12-mCherry-RLuc1 and 10T1/2-GFP-RLuc2 were mixed in a 1:1 ratio, and the mixture was seeded at 100% confluence in 96-well plates (for luciferase assays) and 12-well plates (for imaging). Twelve hours after seeding, the medium was changed to differentiation medium, DMEM with 2% horse serum and 1% antibiotic-antimycotic (Thermo Fisher Scientific, 26050088), to induce myoblast differentiation. DMEM without phenol red (Thermo Fisher Scientific, 21063029) was used in 96-well plates, as phenol red reduced luciferase signal.

Five days after differentiation, cells in 12-well plates were fixed in 4% paraformaldehyde in PBS, permeabilized in 0.3% Triton X-100 for 15 minutes, and stained with Hoechst 33342 (Thermo Fisher Scientific, H3570). Cells in 96-well plates were used for luciferase assays. A cell-permeable firefly luciferase substrate, ViviRen (Promega, E6491), was used per the provider’s instructions. CLARIOstar plate reader (BMG Labtech) was used for luminescence readings.

### Statistics.

Statistical comparisons between groups were evaluated by unpaired and 2-sided Student’s *t* test. For multiple comparisons, 1-way ANOVA was used. A *P* value lower than 0.05 was considered statistically significant.

### Study approval.

For human genetic studies, informed consent forms for the publication of clinical and genetic data were signed by the parents and made part of the digital health care records at the University Medical Center Utrecht. All animal procedures were approved by the Institutional Animal Care and Use Committee at the University of Texas Southwestern Medical Center.

## Author contributions

ARM, YZ, NL, RBD, RHVJ, and ENO wrote and edited the manuscript. RHVJ supervised clinical research and performed project administration and conceptualization. MJVDB, IC, RJJVE, and NGJ performed clinical assessment and data collection. ARM, YZ, and ENO designed the experiments and analyzed the data. FC and ACC designed base editing experiments. ARM, YZ, and CRC performed the experiments. JRM performed the zygote injections to generate *Mymx* R46* mutant mice. MPGM and RHVJ performed human genetic analysis. MGE and LWVO performed identity-by-descent analysis.

## Supplementary Material

Supplemental data

## Figures and Tables

**Table 1:**
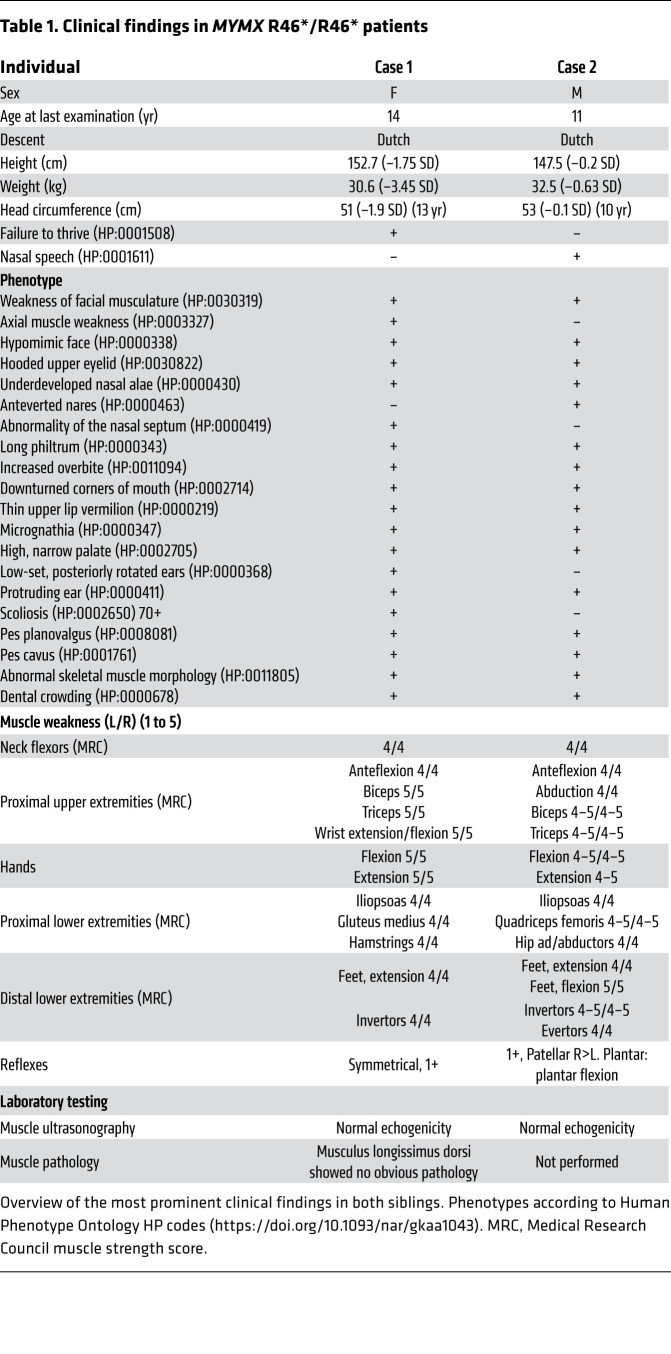
Clinical findings in *MYMX* R46*/R46* patients

## Version 1 06/01/2022

Electronic publication
