# Supplementary material for: Impaired activity of the fusogenic micropeptide Myomixer causes myopathy resembling Carey-Fineman-Ziter syndrome
Source: J Clin Invest. 2022 Jun 1;132(11):e159002. doi: 10.1172/JCI159002 (PMC9151691; doi:10.1172/JCI159002)
Supplement: Supplemental data [file jci-132-159002-s085.pdf]

Impaired activity of the fusogenic micropeptide Myomixer causes myopathy resembling Carey-Fineman-Ziter syndrome

Andres Ramirez-Martinez[1], Yichi Zhang[1*], Marie-Jose van den Boogaard[2*], John McAnally[1], Cristina Rodriguez- Caycedo[1], Andreas C. Chai[1], Francesco Chemello[1], Maarten PG Massink[2], Inge Cuppen[3], Martin G Elferink[2], Robert JJ van Es[4], Nard G Janssen[4], Linda Walraven-van Oijen[2], Ning Liu[1], Rhonda Bassel-Duby[1], Richard H. van Jaarsveld[2**], and Eric N. Olson[1**]

[1] Department of Molecular Biology and Hamon Center for Regenerative Science and Medicine, University of Texas Southwestern Medical Center; Dallas, TX, 75390.

[2] Department of Genetics, University Medical Center Utrecht, The Netherlands.

[3] Department of Neurology, University Medical Centre Utrecht, Utrecht, The Netherlands.

[4] Department of Oral and Maxillofacial Surgery, University Medical Center Utrecht, Utrecht, the Netherlands.

*These authors contributed equally to the work

****Corresponding authors:** Eric N. Olson, Ph.D., University of Texas Southwestern Medical Center, 5323 Harry Hines Boulevard, Dallas, Texas USA 75390-9148

Email: Eric.Olson@utsouthwestern.edu; Phone: 214-648-1187

Richard H van Jaarsveld, Ph.D., University Medical Centre Utrecht, Heidelberglaan 100, 3584CX, Utrecht, the Netherlands. Email: r.h.vanjaarsveld@umcutrecht.nl; Phone: +31(0)88 75 538 00

Supplemental Figure

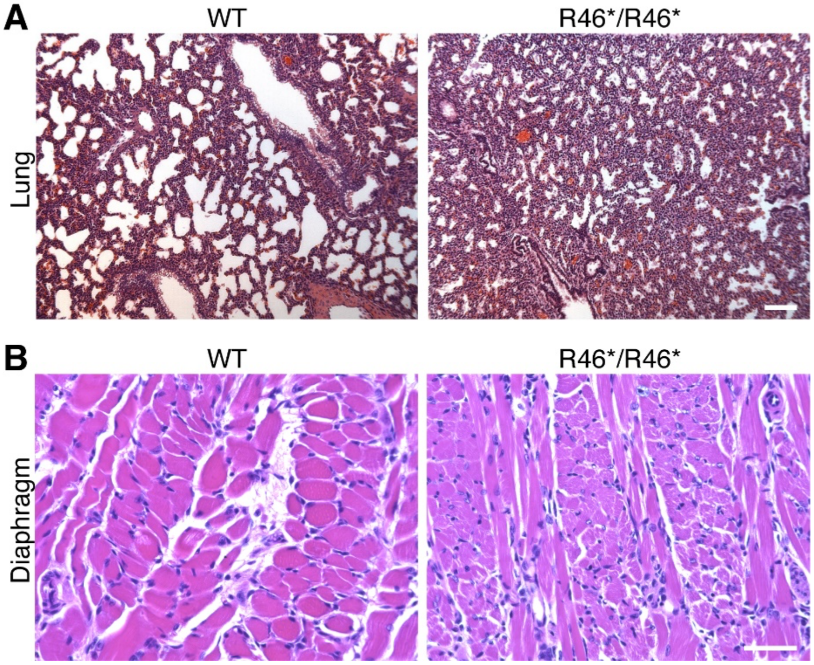

Supplemental Figure 1. A. Hematoxylin and eosin staining of WT and *Mymx* R46*/R46* lungs at birth. Scale bar: 100 μm. **B**. Hematoxylin and eosin staining of WT and *Mymx* R46*/R46* diaphragm at postnatal day 14. Scale bar: 50 μm.

Supplemental clinical report

Case 1

A 14-year-old female presented for care with the chief complaint of weakness and decreased exercise tolerance. The patient was born following an uneventful full-term delivery at 41 weeks, however her postnatal course was complicated by low birth weight (2805g, <3rd percentile), short stature, and failure to thrive. Her parents reported significant feeding difficulties. At the time, the patient underwent extensive testing blood and urine testing for metabolic syndromes which were within normal limits, as well as genetic testing for Silver Russell Syndrome (analysis of UPD7/UPD14, and methylation of H19/LIT1) which was negative. The child went on to have normal motor development.

At the age of 9, the patient reported a significant decline in endurance and reported overall weakness that was progressive along with failure to thrive. Since that time, the patient developed progressive and severe scoliosis, which recently required surgical correction at the age of 13. The patient endorses progressive weight loss and consequent severe malnutrition that ultimately required feeding tube placement. She is otherwise cognitively normal and is attending high school, without other challenges or medical issues except the aforementioned. Family history is significant for a brother with similar postnatal growth challenges, facial dysmorphism, and weakness (see Case 2).

On presentation, the patient was found to be severely underweight with a BMI of 13 at <1st percentile for age. General physical exam findings were notable for significant facial dysmorphism and severe scoliosis. Facial dysmorphic findings included hypotonic and hypomimic facies with hooded and downturned eyes, low-set posteriorly rotated ears, elongated philtrum with a thin vermillion, micrognathia, and pes cavus and pes planovalgus. Targeted neurologic examination uncovered muscular weakness noted as 4/5 on bilateral neck flexors, 4/5 anteflexion in bilateral upper extremities, 4/5 bilateral hip flexors, 4/5 bilateral hamstrings, and 4/5 bilateral plantar flexors and extensors. Neurological examination was non-focal. Radiographic studies confirmed severe scoliosis, micrognathia, and dental crowding. Muscle ultrasonography demonstrated normal echogenicity. Muscle biopsy from the longissimus dorsi was without obvious pathology.

Based on the patient's facial features, muscular weakness, and overall clinical picture, the diagnosis of Carey-Fineman-Ziter Syndrome was considered. Exome sequencing with specific analysis of the genes *TMEM8C (MYMK), ATAC3,* and *BMP2* showed no pathogenic variant; however, analysis of *MYMX* revealed a homozygous C to T variant resulting in conversion of Arg46 to a termination codon in codon 46. SNP array analysis showed a normal female array profile. Both parents were confirmed to be heterozygous carriers of this single nucleotide variation, and the patient's brother (Case 2) was confirmed homozygous for *MYMX* R46*.

Case 2

An 11-year-old boy presented for care with the chief complaint of tired legs. The patient was born following an uneventful full term (41+5) delivery. Postnatal course was complicated by feeding issues from birth, requiring frenotomy prior to initial discharge, and subsequent hospitalization for failure to thrive at three months of age. The child went on to have grossly normal motor development and was able to walk independently at 16 months. Of note, speech development was delayed, and the patient required speech therapy from the age 2 onward; a diagnosis of velopharyngeal insufficiency was made, which ultimately required surgical correction at the age of 4.

The patient predominantly complains of “tired legs” and fatigue despite sleeping 12 hours a night. He denies other complaints. He initially attended special education due to speech delays, but now attends regular primary school and is cognitively normal, without other challenges or medical issues except the aforementioned. Family history is significant for a sister with failure to thrive, facial dysmorphism, severe scoliosis, and muscle weakness (see Case 1).

On presentation, the patient was found to be of normal weight and stature for stated age. General physical exam findings were notable for facial dysmorphism and nasal speech. Facial dysmorphic findings included hypotonic and hypomimic facies with hooded and downturned eyes, anteverted nares, elongated philtrum with a thin vermillion, micrognathia, and pes cavus and pes planovalgus. Neurological examination demonstrated muscular weakness, with 4/5 strength in bilateral neck flexors, 4/5 anteflexion and abduction in bilateral upper extremities, 4-5/5 biceps/triceps, 4/5 bilateral hip flexors, 4-5/5 bilateral quadriceps, and 4/5 bilateral plantar extensors. Neurological examination was non-focal. Muscle ultrasonography demonstrated normal echogenicity.

Given identification of the *MYMX* R46* homozygous mutation in his first-degree sibling (Case 1) who demonstrated more pronounced pathological features, analysis of the *MYMX* gene in the patient confirmed the same homozygous mutation.
